# Metabolic Outcomes of Anaplerotic Dodecanedioic Acid Supplementation in Very Long Chain Acyl-CoA Dehydrogenase (VLCAD) Deficient Fibroblasts

**DOI:** 10.3390/metabo11080538

**Published:** 2021-08-13

**Authors:** Igor Radzikh, Erica Fatica, Jillian Kodger, Rohan Shah, Ryan Pearce, Yana I. Sandlers

**Affiliations:** Department of Chemistry, Cleveland State University, Cleveland, OH 44115, USA; i.radzikh@vikes.csuohio.edu (I.R.); e.m.fatica@csuohio.edu (E.F.); j.kodger@vikes.csuohio.edu (J.K.); r.r.shah22@vikes.csuohio.edu (R.S.); r.pearce52@vikes.csuohio.edu (R.P.)

**Keywords:** VLCAD deficiency, dodecandioic acid, acylcarnitines, Krebs cycle

## Abstract

Very long-chain acyl-CoA dehydrogenase deficiency (VLCADD, OMIM 609575) is associated with energy deficiency and mitochondrial dysfunction and may lead to rhabdomyolysis and cardiomyopathy. Under physiological conditions, there is a fine balance between the utilization of different carbon nutrients to maintain the Krebs cycle. The maintenance of steady pools of Krebs cycle intermediates is critical formitochondrial energy homeostasis especially in high-energy demanding organs such as muscle and heart. Even-chain dicarboxylic acids are established as alternative energy carbon sources that replenish the Krebs cycle by bypassing a defective β-oxidation pathway. Despite this, even-chain dicarboxylic acids are eliminated in the urine of VLCAD-affected individuals. In this study, we explore dodecanedioic acid (C12; DODA) supplementation and investigate its metabolic effect on Krebs cycle intermediates, glucose uptake, and acylcarnitine profiles in VLCAD-deficient fibroblasts. Our findings indicate that DODA supplementation replenishes the Krebs cycle by increasing the succinate pool, attenuates glycolytic flux, and reduces levels of toxic very long-chain acylcarnitines.

## 1. Introduction

Very long-chain acyl-CoA dehydrogenase deficiency (VLCADD, OMIM 609575) is manifested by a wide range of clinical phenotypes including hypertrophic and dilated cardiomyopathy, rhabdomyolysis, myopathy, and hypoglycemia [1,2,3,4]. Some of these symptoms can be ameliorated by nutritional restrictions. Current dietary strategies are primarily focused on eating frequent meals and avoiding consumption of very long-chain fatty acids, instead offering carbohydrates and medium-chain triglycerides as the energy source [5]. Despite the special diet, nutritional management has limited success to treat some symptoms and metabolic derangements in VLCADD affected individuals.

VLCAD deficiency leads to a defect in long-chain fatty acids catabolic pathway with subsequent disruptions in energy production [6,7] and mitochondrial function [8,9]. In high energy demanding organs such as muscle and heart, carbon energy substrate utilization and the maintenance of adequate pools of Krebs cycle intermediates in mitochondria are especially critical. Disruptions in adequate energy production and mitochondrial bioenergetic pathways are implicated in the risk of development rhabdomyolysis [10] and cardiomyopathy [11]. To target impaired mitochondrial bioenergetics in VLCADD, anaplerotic therapy was proposed as a new treatment strategy [12,13]. This therapeutic approach is directed towards the restoration of energy production by replenishing pools of Krebs cycle intermediates [14] while bypassing defective β-oxidation of long-chain fatty acids (LCFA). Nutritional therapy with a recently FDA-approved anaplerotic triglyceride triheptanoin in VLCADD demonstrated long-term positive effects, such as improvement of some clinical symptoms and hospitalization frequency [15,16,17,18]. Nevertheless, VLCADD presents with a very heterogenic phenotype and clinical outcomes under triheptanoin supplementation are also heterogenic [16,17]. Oral triheptanoin has a limited effect on the peripheral tissues [19] and may induce gastrointestinal symptoms leading to some degree of lipotoxicity. Triheptanoin also does not significantly reduce episodes of rhabdomyolysis, thus the search for the new therapeutic agents is still ongoing.

More recently, to address exercise intolerance and rhabdomyolysis another anaplerotic molecule was investigated. Bleeker et al. demonstrated that ketone ester-induced nutritional ketosis has beneficial outcomes and improves exercise performance in five VLCAD-affected subjects [20]. The data presented in this study suggest that anaplerotic ketone ester increases ATP levels in muscles.

Even-chain dicarboxylic acids (DAs) also exhibit anaplerotic characteristics and have been proposed as an alternative energy substrate [21]. DAs are produced endogenously through the corresponding fatty acid ω-oxidation or from the β-oxidation of longer dicarboxylic acids. In normal circumstances, these pathways are considered minor fates however, when β-oxidation becomes impaired, fatty acids are channeled to produce DAs. DAs (C6-C12) metabolism in mitochondria and peroxisomes, yields a Krebs cycle intermediate and gluconeogenic precursor succinyl-CoA. Due to their anaplerotic effect and high energy density [22], DAs were proposed as an alternative fuel energy substrate in parental nutrition [21,22,23]. It is evident however that overproduction and accumulation of hepatic DA are toxic [24]. Dicarboxylic aciduria is strongly associated with fatty acids oxidation disorders while C8-C12 DAs are eliminated in urine in FAOD-affected individuals [25,26] especially during metabolic instability and fasting episodes.

**Figure 1:**
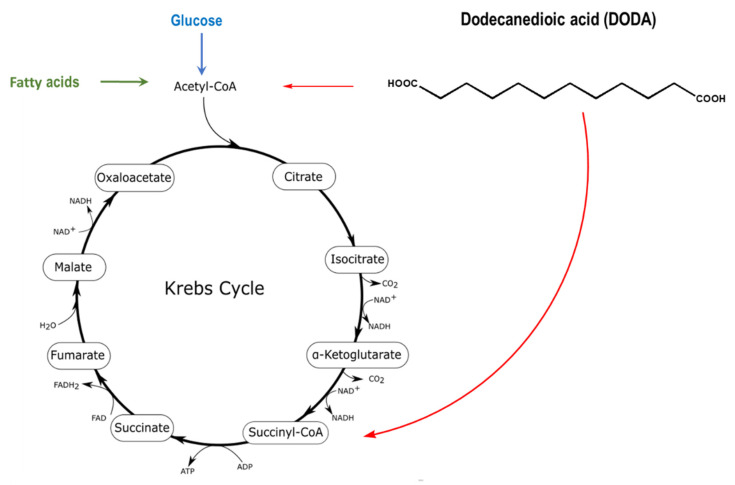
DODA oxidative metabolism in mitochondria and peroxisomes yields acetyl-CoA and succinyl-CoA that can anaplerotically replenish the Krebs cycle (red arrows). The identity of DODA peroxisomal oxidation intermediates transferred from peroxisome to mitochondria for further oxidation is not fully elucidated [31].

Dodecanedioic acid (DODA), a twelve carbon/medium-chain water-soluble DA derives from β-oxidation of longer chain DAs and by the direct ω-oxidation of lauric acid (C14). DODA enters mitochondria through the mitochondrial transport system similar to the corresponding monocarboxylic fatty acids, but does not require a carnitine shuttle [27,28]. Mitochondria (Figure 1) and peroxisomes are major sites of DODA oxidative metabolism [29,30].

Due to its anaplerotic effect, the pharmacokinetics of DODA were studied in humans [32,33,34]. Given its solubility, DODA can be introduced through peripheral veins as an inorganic salt. Following administration, it is immediately available for the tissue energy requirements and exhibits a very low urinary clearance [22,29]. These observations suggest that DODA supplementation can potentially benefit some conditions associated with energy deprivation. Limited data, however, are available on DODA metabolic effect in comparison to the recently FDA-approved triheptanoin (DOJOVI^TM^, Ultragenyx, Novato, CA, USA) (Table 1).

Here, we explore dodecanedioic acid (C12; DODA) supplementation and investigate its metabolic effect on Krebs cycle intermediates, glucose uptake, and acylcarnitine profiles in VLCAD-deficient fibroblasts.

## 2. Results

### 2.1. DODA Cellular Uptake

**Figure 2:**
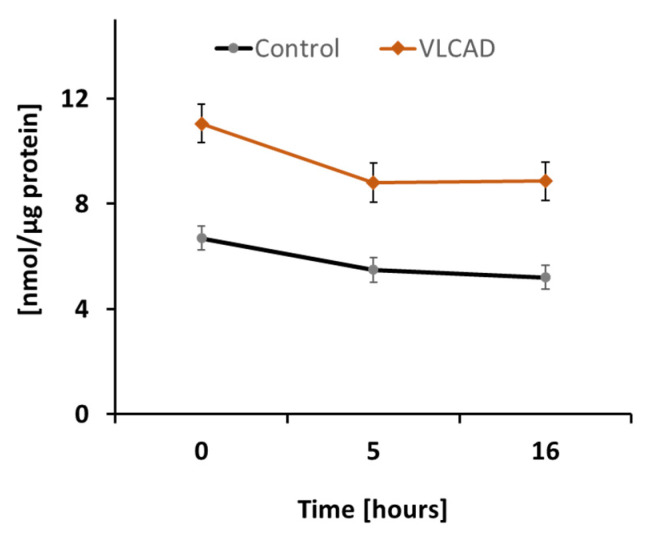
DODA cellular uptake (*n* = 5). DODA level in cellular media is normalized to the total protein amount.

To assess DODA metabolic impact in control and VLCAD deficient cells, we first analyzed DODA cellular uptake. Preliminary studies indicated that 1 mM DODA is the optimum concentration that yields an increase in succinate (Figure S1). In the time period of sixteen hours and the presence of other competing carbon substrates such as 5 mM glucose and 0.2 mM palmitic acid-BSA, we observed a decrease of DODA in culture media by 22.3% and 20.0% in control and VLCAD-deficient fibroblasts, respectively (Figure 2).

### 2.2. Krebs Cycle Intermediate

**Figure 3:**
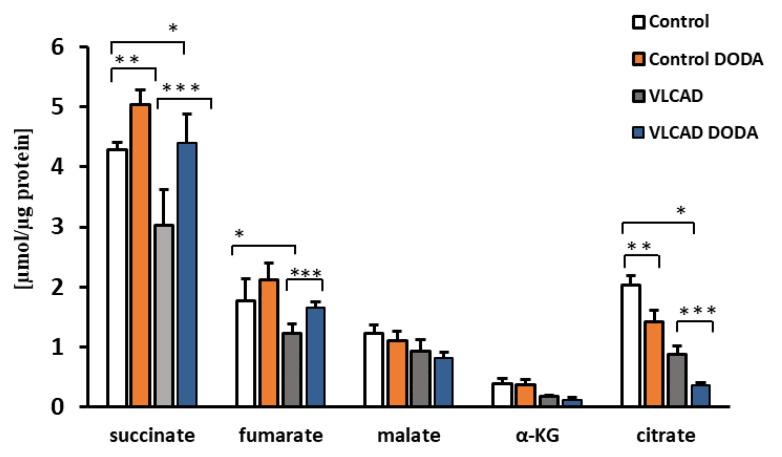
Cellular levels of Krebs cycle intermediates. *n* = 5, * *p* < 0.05 Control vs. VLCAD; ** *p* < 0.05 Control vs. Control DODA; *** *p* < 0.05 VLCAD vs. VLCAD DODA.

At the baseline, VLCAD deficient fibroblasts demonstrated a decrease in succinate, α-KG, and citrate (Figure 3). Low fumarate levels in VLCAD-deficient cells did not achieve statistical significance. Incubation with 1 mM DODA for sixteen hours resulted in alterations of Krebs cycle intermediates profiles. In agreement with the expected anaplerotic DODA characteristics, succinate in both control and VLCAD-deficient cell lines was the most significantly impacted metabolite in comparison to the baseline. A mild increase in fumarate in control cells and a marked decrease in citrate in both control and VLCAD-deficient cells were also detected (Figure 3).

### 2.3. Glucose Uptake and Glycolytic Flux

**Figure 4:**
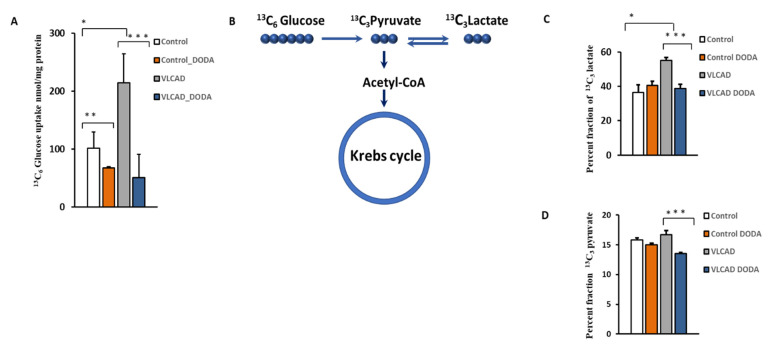
(**A**) Cellular ^13^C_6_-Glucose uptake; (**B**) Schematic ^13^C incorporation; (**C**) Percent fraction of ^13^C_3_-lactate; (**D**) ^13^C_3_-pyruvate; * *p* < 0.05 Control cells vs. VLCADD cells; ** *p* < 0.05 Control cells vs. Control/Control DODA treated; *** *p* < 0.05 VLCADD cells vs. VLCADD cells DODA treated.

The VLCAD phenotype manifests with intermittent hypoglycemia. To determine the effect of 1 mM DODA supplementation on cellular glucose uptake, control and VLCAD-deficient cells were incubated in glucose-free media supplemented with 5 mM ^13^C_6_-glucose, 0.2 mM palmitate-BSA, 0.4 mM l-carnitine, and 1 mM of DODA. Next, we analyzed ^13^C_6_-glucose levels in culture media at time zero (t = 0 h) and after five hours (t = 5 h). In five hours, VLCAD cells consumed a significantly higher amount of glucose (214.6 ± 50.0 nmol/mg protein vs. 101.1 ± 28.9 nmol/mg protein in the control cell). ^13^C_6_-glucose level in cell media five hours post 1 mM DODA loading indicated that cells consumed less glucose with fold changes of −1.50 and −4.24 for control and VLCAD deficient cells, respectively (Figure 4A). We also monitored the incorporation of ^13^C from ^13^C_6_-glucose to the downstream ^13^C_3_-pyruvate and ^13^C_3_-glycolytic lactate (Figure 4C,D). DODA has no effect on ^13^C-labeled cellular pools of pyruvate and lactate in control cells, however, there is a significant reduction of ^13^C_3_-pyruvate and ^13^C_3_ lactate pools (Figure 4C,D) and their ratios (Table 2) in VLCAD-deficient fibroblasts treated with DODA.

### 2.4. Acylcarnitines Profiles

**Figure 5:**
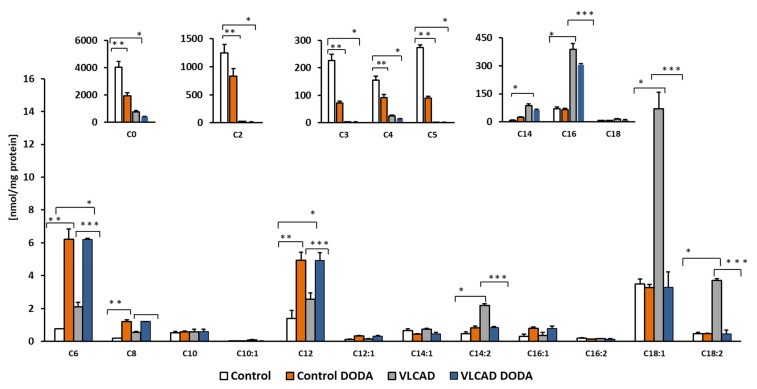
Cellular acylcarnitines levels. Cells were supplemented with a mixture of 0.2 mM oleic, palmitic and linoleic-BSA conjugated fatty acids, *n* = 5, * *p* < 0.05 Control vs. VLCAD; ** *p* < 0.05 Control vs. Control DODA; *** *p* < 0.05 VLCAD vs. VLCAD DODA.

Circulating acylcarnitines are diagnostic markers of incomplete fatty acid oxidation in VLCADD. We supplemented cells with a mixture of oleic, palmitic, and linoleic-BSA conjugated fatty acids and analyzed cellular acylcarnitines at the baseline and post-incubation with 1 mM DODA. DODA supplementation induces changes in acylcarnitine profiles (Figure 5). The decrease in the total amount of long-chain acylcarnitines in VLCAD-deficient cells was significant (Table 2), while the overall impact on long-chain acylcarnitine levels in control cells was mild.

## 3. Discussion

Very long-chain acyl-CoA dehydrogenase (VLCAD) deficiency, a metabolic disorder of long-chain mitochondrial fatty acids (C14-C20) oxidation, is strongly associated with energy deficit and accumulation of toxic fatty acids oxidation intermediates. The disorder is screened as a part of the newborn screening (NBS) program based on dried blood spot acylcarnitine profiles with a follow-up second-tier testing such as urine organic acids and molecular confirmatory tests. The defective very long-chain acyl-CoA dehydrogenase enzyme is a key protein in mitochondrial energy metabolism, thus energy deficit and perturbed substrate utilization play a significant role in disease pathophysiology. Disease treatment primarily focuses on the management of clinical symptoms. Nutritional management focuses on restricting intake of dietary fat while supplementing patients with medium-chain triglycerides (MCT oil) in an attempt to restore energy deficit and Krebs cycle substrates balance.

A more recent therapeutic approach using anaplerotic supplements for fatty acid oxidation disorders and other inherited metabolic diseases has been proposed [38,39]. These anaplerotic energy sources contribute to energy production by increasing the availability of Krebs cycle intermediates or their precursors (acetyl-CoA and propionyl-CoA) required for optimal energy production while bypassing the defective metabolic pathways [37]. For instance, triheptanoin (DOJOLVI^TM^), an anaplerotic C7-triglyceride was recently approved by FDA for LC-FOAD management. Medium even-chain dicarboxylic acids (C6-C12) also exhibit anaplerotic characteristics and effectively replenish Krebs cycle intermediates with efficient energetic values [21,22].

In the present study, we explored the metabolic outcomes of 1 mM DODA supplementation on Krebs cycle intermediates, acylcarnitines, and glucose uptake in fibroblasts derived from healthy and documented VLCAD-deficient individuals.

To encourage competitive uptake of DODA, we cultured cells in a state of low glucose (5 mM) and low palmitate-BSA (0.2 mM). Under these conditions, both control and VLCAD-deficient fibroblasts consumed DODA at a similar rate, indicating that 364 A>G mutation does not affect DODA cellular uptake (Figure 2). A number of earlier studies explored DODA metabolism and reported that DODA catabolism yields succinate [35]. More recently, Jin et al. perfused rat liver with ^13^C-labeled DODA and detected ^13^C labeling in Krebs cycle intermediates, short-chain CoAs, and β-hydroxybutyrate. The study provides solid evidence that DODA catabolism yields anaplerotic acetyl-CoA and succinyl-CoA in the liver [31]. Under our experimental conditions, acyl-CoA pools were under the limit of detection in fibroblasts however, we analyzed cellular Krebs cycle intermediates (Figure 3). At the baseline, VLCAD deficient cells demonstrated decreased levels of succinate, α-KG, and citrate (Figure 3). A depletion of Krebs cycle intermediates that was recently also reported in VLCAD (−/−) mice [40] and eight VLCAD patients [18] contributes further to energy-related impairments associated with the VLCAD phenotype.

In agreement with the anaplerotic effect of DODA reported by others, 1 mM DODA loading resulted in an increase in succinate level in both control and VLCAD-deficient cells by 1.17 and 1.45 fold changes, respectively. A similar fold change increase was also observed in cellular fumarate levels. These findings support DODA anaplerotic activity. A marked reduction in cellular citrate in both control and VLCAD-deficient fibroblasts under DODA supplementation is consistent with the citrate synthase inhibition by the succinyl-CoA [41] produced from DODA. A marked reduction in cellular citrate in both control and VLCAD-deficient fibroblasts under DODA supplementation can be possibly explained by the succinyl-CoA inhibitory effect on citrate synthase. Tucci et al. highlight that citrate synthase activity response is tissue-specific [42]. The study reports a decrease in wild-type mice heart citrate synthase activity under anaplerotic triheptanoin diet with no change in enzyme activity in VLCAD-deficient mice heart. At the same time, there is an increase in citrate synthase activity in the liver for both wild-type and VLCAD genotypes. Although triheptanoin is metabolized differently from DODA, it similarly results in succinyl-CoA production. Despite the prospect that succinyl-CoA produced from DODA may lead to the decrease of the total cellular citrate pool, high succinyl-CoA is converted to the succinate and may increase the respiratory activities of the electron transport chain and thus have an overall positive impact on VLCAD deficiency-induced overall mitochondrial dysfunction. Indeed, we found that the lactate/pyruvate ratio that represents a clinical, marker of mitochondrial function and oxidative metabolism is normalized in VLCAD deficient cells post DODA treatment (Table 3).

Intermittent hypoglycemia is one of the clinical manifestations of fatty acid oxidation disorders. In VLCADD, the inability to oxidize fatty acids leads to hypoketotic hypoglycemia due to a deficit in ketones as an energy source and an increase in glucose uptake by the peripheral tissues [43]. In comparison to the control cells, VLCAD-deficient fibroblasts demonstrate a significantly higher baseline ^13^C_6_-glucose uptake (Figure 4), providing evidence of metabolic adaptation to the VLCAD deficiency. At the same time, cellular levels of ^13^C_3_-pyruvate in VLCAD fibroblasts were also mildly elevated suggesting that induced glycolytic flux contributes to the excessive glucose uptake. A marked increase in cellular ^13^C_3_ lactate levels is a biochemical hallmark of mitochondrial dysfunction. It also provides evidence of an increased compensatory rate of anaerobic glycolysis to meet energy demands at the time that VLCFA utilization is restricted. An increase in lactate in our study is in contrast to a study by Ventura et al. [44] which reported a normal lactate/pyruvate ratio in VLCAD-deficient fibroblasts. The discrepancy in the ratios can be explained by the heterogeneous presentation of VLCADD associated with different mutations. 1 mM DODA loading did not significantly affect ^13^C_3_-lactate and ^13^C_3_-pyruvate levels in control cells (Figure 4 and Table 3). The observed increase in ^13^C_3_-lactate/^13^C_3_-pyruvate in control cells post DODA incubation from 2.31 to 2.70 is statistically insignificant as a result of a large standard deviation in ^13^C_3_-lactate levels (Figure 4). In VLCAD-deficient cells, DODA led to a decrease in lactate/pyruvate ratio that can be attributed to the attenuation of glycolytic flux as evident by reduced glucose uptake and a decrease in the downstream glycolytic ^13^C_3_-pyruvate.

Accumulation of circulating long-chain acylcarnitines is a biochemical diagnostic hallmark of VLCADD. In humans, circulating levels of acylcarnitines reflect tissue pools, and an increase in long and long-chain acylcarnitine pools is associated with processes that are detrimental to cardiac work [45,46,47]. VLCAD-deficient cells supplemented with a mixture of fatty acids exhibit a significant accumulation of C14 and C14:2 (Figure 5) in agreement with the VLCAD phenotype. Affected fibroblasts also demonstrated a marked decrease in short-chain acylcarnitines C3 and C5 which is consistent with a decrease in branched-chain amino acid utilization (BCAA). C4 acylcarnitine that derives from amino acids and lipid metabolism was also markedly decreased. After incubation with 1mM DODA for sixteen hours in VLCAD deficient cells, we observed a decrease in some short-chain acylcarnitines (Figure 5) and a total concentration of long-chain acylcarnitines (C14-C18) including unsaturated species (Table 2) with the most significant changes in C14:2, C18:1 and C18:2 (Figure 5). In contrast, in control cells, DODA impact on long-chain acylcarnitines is not significant, however, alterations in short-chain species (C3–C5) are similar to the VLCAD deficient cells. The etiology of free carnitine decrease in control fibroblast post-DODA addition is not clear at this moment. Intracellular C12DC carnitine was not detected, so it is unlikely that DODA binds free carnitine and depletes carnitine pool however, we didn’t analyze acylcarnitines that possibly eliminated to the cell media. Some anticancer drugs (etoposide, actinomycin D, and vinblastine) and antibiotics (cephaloridine, cefepime, and cefluprenam, levofloxacin, and grepafloxacin) can inhibit carnitine transporter OCTN2 leading to secondary carnitine deficiency [48,49]. The clinical manifestation of the secondary carnitine deficiency was not reported in human studies with DODA. In fact, DODA was suggested as a component of parental nutrition [22,32,50]. It is established that DODA is first shortened in peroxisomes followed by final oxidation steps inside the mitochondria [30,31]. Once produced in peroxisomes, medium-chain acyl-CoAs are transported for further oxidation in the mitochondria through the carnitine shuttle [31,51]. The mechanism of the medium-chain acylcarnitines increase in cells post DODA treatment is unclear at the moment. DODA feeding in mice induces expression of peroxisomal FAO enzymes and MCAD/LCAD genes expression, suggesting induced production of medium-chain acylcarnitines. More studies with stable isotope tracers are needed to clarify the increase in medium-chain C6–C12 acylcarnitines in our cellular model. Overall, the observed acylcarnitines pattern in VLCAD deficient cells indicates that through anaplerotic acyl-CoA production, DODA loading modifies substrate preferences, reducing utilization of BCAA and VLCFA as carbon substrates.

While our data demonstrate DODA metabolic impact in VLCAD deficient fibroblasts these findings however are presented in vitro cellular model. Dicarboxylic acidemia in VLCAD deficiency is a result of compensatory and protective mechanisms where a non-metabolized VLCFAs are channeled towards ω-oxidation. Once generated through ω-oxidation mainly in the liver and kidney, DAs are activated to the corresponding CoAs and are degraded through β-oxidation by peroxisomal and mitochondrial enzymes [30,52,53,54]. During fasting and metabolic instability, this protective mechanism is upregulated as fasting induces CYP4A ω-hydroxylases and acyl CoA thioesterases (ACOTs) involved in synthesis and degradation of DAs, respectively [55]. Indeed, when the rate of DA production during metabolic decompensation exceeds tissue catabolic capacities, DAs accumulate in the blood and consequently will be eliminated in urine. The metabolic effect of DODA is also likely to be tissue-specific. Nevertheless, DODA is an alternative carbon substrate, it is not clear to what extent the most compromised VLCAD deficient tissues such as muscle and heart are capable to uptake it. In our study, fibroblasts consumed only twenty-one percent of DODA (mean of control and VLCAD deficient cells) during a sixteen-hour experiment, although Salinari et al. report that following DODA oral administration in diabetes type 2 subjects the total tissue uptake is forty-seven percent [56].

The perspective and the clinical significance: The defect in VLCFA oxidation underlies the metabolic inflexibility and aberrant energy metabolism in the heart and muscle leading to the clinical manifestation of VLCAD deficiency. Alternative energy carbon substrates improve cardiac symptoms and muscle energy balance during exercise performance. Dodecanedioic acid is an alternative carbon energy substrate that bypasses the defective pathway in the course of β-oxidation and exhibits high caloric energy density. Our study reveals that 1 mM DODA intervention leads to a decrease in glucose uptake, normalizes lactate/pyruvate ratio, and reduces some long-chain acylcarnitines. Taken together, our findings indicate that DODA potentially can ameliorate VLCAD symptoms, however, additional studies are needed to elucidate DODA fate in muscle and heart as well as its impact on metabolism during exercise.

Study limitations: VLCAD deficiency has a wide spectrum of clinical manifestation and heterogenic phenotypic variation. Our proof-of-principle study is focused on one single mutation. We predict that DODA metabolic response is mutation dependent whereas further studies are needed to fully assess DODA metabolic impact with more VLCADD-deficient fibroblasts and matching control cell lines

## 4. Materials and Methods

### 4.1. Cells

Human control and VLCAD-deficient fibroblasts (364 A>G) were obtained from Coriell Institute (control GM08680 5-month-old at sampling, disease GM17475, 10 days old at sampling). Based on the provided characteristics, the patient has severe early onset of the disease, presenting with cardiomyopathy and hypoglycemia. The patient is homozygous for an A>G transition at nucleotide 364 of the ACADVL gene resulting in an asparagine to aspartic acid change at codon 122 in the precursor protein [Asn122Asp (N122D)]; Approval of the Institutional Review Board was not required upon purchase. The material transfer agreement was approved by Cleveland State University institutional board. Fibroblasts were grown in 5 mM glucose DMEM containing 10% FBS, 100 mg/mL penicillin, 0.4 mM l-carnitine, and 0.2 mM linoleic, oleic, and palmitic acids BSA conjugated at 37 °C in 5% CO_2_. For the treatment experiments, DODA was added to the cell media at a final concentration of 1 mM. This concentration was determined empirically as the optimal concentration for the anaplerotic effect in our system.

### 4.2. Dodecanedioic Acid Uptake Assay

To the 20 µL aliquot of cell media, 10 µL of undecanedioic acid (1 mM) was added (as an internal standard) followed by the addition of 0.3 µL of 3N HCl and 300 µL of ethyl acetate. Samples were vortexed and centrifuged at 15,000 rpm for 10 min. An aliquot (200 µL) of the upper organic phase was transferred to a new tube and dried at room temperature under nitrogen, followed by derivatization with 100 µL of N,O-Bis(trimethylsilyl)trifluoroacetamide (MilliporeSigma, Burlington, MA, USA) at 90 °C for 30 min. Samples were injected onto GCMS (Agilent, Santa Clara, CA, USA) operated in electron impact (EI) mode- single ion monitoring (SIM) mode with *m*/*z* 359 and *m*/*z* 345 target ions for dodecanedioic (Table S1) and undecanedioic acids, respectively.

### 4.3. Glucose Uptake Assay

Glucose uptake was assayed as described previously [57]. More details can be found in supplemental materials.

### 4.4. Krebs Cycle Intermediates, Cellular Lactate, and Pyruvate

Cells were washed two times with cold Dulbecco’s phosphate-buffered saline (DPBS) followed by one cold water wash. Metabolism quenching was achieved by the addition of cold methanol/5% acetic acid. Cells were scraped, vortexed, then centrifuged at 5000 rpm for 15 min. Cell lysates were separated, dried, and derivatized with methoxyamine (Millipore Sigma) in pyridine (20 mg/mL, 40 μL, 80 °C for 60 min) followed by N-methyl-N-tert-butyldimethylsilyltrifluoroacetamide (MilliporeSigma 60 μL, 70 °C for 45 min). Krebs cycle intermediates were analyzed by EI-GCMS. Target ions and retention times can be found in supplemental materials (Table S1).

### 4.5. Acylcarnitine Analysis

Cells were washed two times with cold Dulbecco’s phosphate-buffered saline (DPBS) followed by one cold water wash. Metabolism quenching was achieved by the addition of cold methanol/5% acetic acid then 100 μL of internal standard solution (Cambridge Isotopes, Tewksbury, MA, USA, NSK B, working solution) was added. Samples were dried under a nitrogen stream at room temperature, followed by derivatization with 60 μL of 3 N HCl/n-butanol for 45 min at 65 °C. The derivatized samples were dried and reconstituted in mobile phase B. LC-MS/MS analysis was carried out with a SCIEX 5500 QTrap mass spectrometer operated in +ESI/multiple reaction monitoring scans (MRM). Chromatography and mass spectrometry method details can be found in supplemental materials (Tables S2–S4).

### 4.6. Statistical Analysis

Relative metabolites levels or percent fraction of ^13^C labeled metabolite are given as mean ± SD. There were five biological replicates for each condition. Significance was tested with Student’s *t*-test. A difference of *p* ≤ 0.05 was considered significant.

## Figures and Tables

**Table 1:** Biochemical and anaplerotic characteristics of triheptanoin and DODA.

Triheptanoin	DODA
Not water soluble Metabolized in mitochondria Anaplerosis: oxidized to acetyl-CoA and propionyl-CoA Induce ketogenesis (C5 and C4 ketone bodies) Effect on glucose metabolism: significantly decreases hypoglycemia episodes in VLCAD affected individuals [35] Energy characteristics: 8.3 kcal/mL (DOJOLVI^TM^ (triheptanoin) oral liquid data from www.fda.gov (accessed on 29 June 2020)) Effect on Krebs cycle intermediates: increase plasma citrate, aconitate, fumarate, and malate in VLCAD affected individuals [18] Effect on lipids profile: increase in odd chain sphingomyelins, phosphatidylcholines, and phosphatidylethanolamines in VLCAD affected individuals [18]	Water-soluble Metabolized in peroxisomes and mitochondria Anaplerosis: oxidized to acetyl- CoA and succinyl-CoA [22,31,36] Does not induce ketogenesis Effect on glucose metabolism: induces a sparing effect on whole-body glucose uptake, in non-insulin-dependent diabetes mellitus [23] Energy characteristics: 7.2 kcal/g [22] Effect on Krebs cycle intermediates: increases succinate levels [22,31,37] Effect on lipids profile: unknown

**Table 2:** Total long-chain (saturated and unsaturated C14-C18) cellular acylcarnitines.

*n* = 5	Total Long Chain Acyl Carnitines (nmol/mg Protein)
Control	91
VLCAD	511
Control + DODA	103
VLCAD + DODA	397

**Table 3:** ^13^C_3_ lactate/^13^C_3_ pyruvate ratio (*n* = 5).

Cell Type	^13^C_3_ Lactate/^13^C_3_ Pyruvate
Control cells	2.31
VLCADD cells	3.31
Control cells/1 mM DODA	2.70
VLCADD cells/1 mM DODA	2.86

## Data Availability

All data are available within the manuscript and in supplemental materials.

## Supplementary Materials

Supplementary materials can be found at https://www.mdpi.com/2218-1989/11/8/538/s1. Figure S1: Optimization of DODA concentration. Change in succinate level in normal fibroblasts in different DODA concentrations (6 h), Figure S2: DODA EI-GCMS spectrum as trimethylsilyl derivative, Figure S3: Total ion chromatogram. EI-GCMS, Table S1: Target ions as tert-Butyltrimethylsilyl derivatives, Table S2: LC gradient program for acyl carnitines analysis, Table S3: Acylcarnitines mass spectrometry parameters, Table S4: Acylcarnitines retention times.

## Abbreviations

DODA	Dodecanedioic acid
DA	Dicarboxylic acid
α-KG	α-Ketoglutarate
VLCFA	Very long-chain fatty acids
VLCADD	Very long-chain acyl-CoA dehydrogenase deficiency
LC-FAOD	Long-chain fatty acid oxidations disorders
