# Supplementary material for: Metabolic Outcomes of Anaplerotic Dodecanedioic Acid Supplementation in Very Long Chain Acyl-CoA Dehydrogenase (VLCAD) Deficient Fibroblasts
Source: Metabolites. 2021 Aug 13;11(8):538. doi: 10.3390/metabo11080538 (PMC8412092; doi:10.3390/metabo11080538)
Supplement: Supplementary file 1 [file metabolites-11-00538-s001.zip › metabolites-1206186-supplementary.pdf]

Cellular Krebs cycle intermediates, lactate and pyruvate

GC-MS analysis was carried out with a 5977 GC-MS (Agilent) operated in EI mode and equipped with an HP-5ms column (Agilent). Retention times and fragmentation mass spectra of all metabolites were confirmed with commercially available standards and the NIST library. Unlabeled (M0) ions monitored through the method are presented in Table S1. Oven program as following: initial oven temperature set at 80°C, hold for 3 minutes, then ramp 15°C/minute up to 305°C and hold for 3 minutes.

Name	m/z M0	m/z M+3 (^{13}C)	Retention Time (min)
Succinate	289	-	11.567
Fumarate	287	-	11.758
Malate	419	-	13.967
α-Ketoglutarate	346	-	13.188
Citrate	459	-	16.771
Lactate	261	264	9.479
Pyruvate	174	177	7.392
Tricarboxylic Acid (as internal standard)	461	-	15.662

Table S1: Target ions as tert-Butyltrimethylsilyl derivatives.

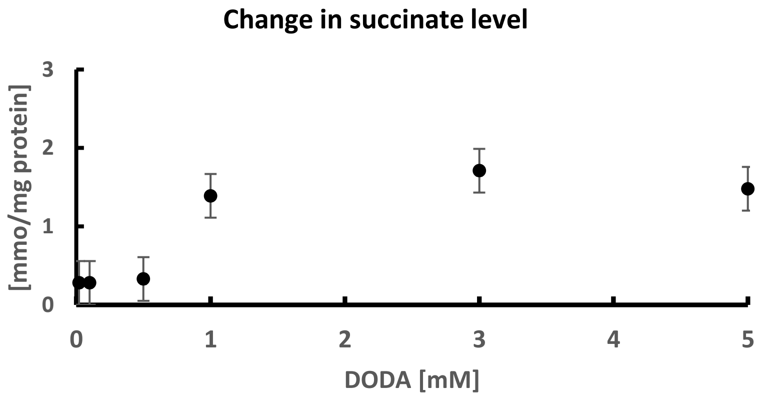

Figure S1: Optimization of DODA concentration. Change in succinate level in normal fibroblasts in different DODA concentrations (6 hours).

Acylcarnitines analysis:

LC-MS/MS for acyl carnitines

ESI source temperature 700^0C, ion spray voltage + 4500V. Column Waters Xbridge BEH C18 (130Å, 2.5 µm, 2.1 mm x 75 mm). Mobile phase A: 0.1 % formic acid in water; mobile phase B: 0.1 % formic acid in acetonitrile. Injection volume 1 µl. Gradient program:

Time (min)	Percent of mobile phase B
**0**	20
**1**	20
**8**	37
**22**	100
**26**	100
**26.1**	20
**30**	20

Table S2. LC gradient program for acyl carnitines analysis

ID	Q1 Mass (Da)	Q3 Mass (Da)	Dwell (msec)	DP	EP	CE	CXP
**C0**	218	85	15	100	2	20	10
**C0 ISTD**	227	85	15	100	2	20	10
**C2**	260	85	15	100	2	20	10
**C2 ISTD**	263	85	15	100	2	20	10
**C3**	274	85	15	100	2	20	10
**C3 ISTD**	277	85	15	100	2	20	10
**C4**	288	85	15	100	2	20	10
**C4 ISTD**	291	85	15	100	2	20	10
**C5**	302	85	15	100	10	20	10
**C4-OH**	304	85	15	100	2	20	10
**C5 ISTD**	311	85	15	100	10	20	10
**C6**	316	85	15	100	10	20	10
**C6-OH**	332	85	15	100	10	20	10
**C8**	344	85	15	100	8	25	10
**C8 ISTD**	347	85	15	100	8	25	10
**C3-DC**	360	85	15	100	2	20	10
**C12:1**	398	85	15	100	8	25	10
**C12**	400	85	15	100	8	25	10
**C12:1-OH**	414	85	15	100	8	25	10

**C12-OH**	416	85	15	100	8	25	10
**C14:2**	424	85	15	100	2	30	10
**C14:1**	426	85	15	100	2	30	10
**C14**	428	85	15	100	2	30	10
**C14 ISTD**	437	85	15	100	2	30	10
**C14-OH**	444	85	15	100	2	30	10
**C16:2**	452	85	15	100	2	30	10
**C16:1**	454	85	15	100	2	30	10
**C16**	456	85	15	100	2	30	10
**C16 ISTD**	459	85	15	100	2	30	10
**C16-OH**	472	85	15	100	2	30	10
**C18:2**	480	85	15	100	2	30	10
**C18:1**	482	85	15	100	2	30	10
**C18**	484	85	15	100	2	30	10
**C22**	510	85	15	100	2	30	10

Table S3. Acylcarnitines mass spectrometry parameters.

Analyte Peak Name	Retention Time
**C0**	0.64
**C2**	1.18
**C3**	2.19
**C4**	3.47
**C5**	5.09
**C4-OH**	5.00
**C6**	7.27
**C6-OH**	8.77
**C8:1**	9.11
**C8**	10.34
**C3-DC**	3.02
**C4DC**	4
**C12:1**	13.43
**C12**	14.08
**C12-OH**	14
**C14:2**	15.09

**C14:1**	14.90
**C14**	15.72
**C14-OH**	15.50
**C16:1**	16.01
**C16**	17.11
**C16-OH**	15.50
**C18:2**	16.39
**C18:1**	17.30
**C18**	18.29
**C22**	21.23

Table S4. Acylcarnitines retention times.

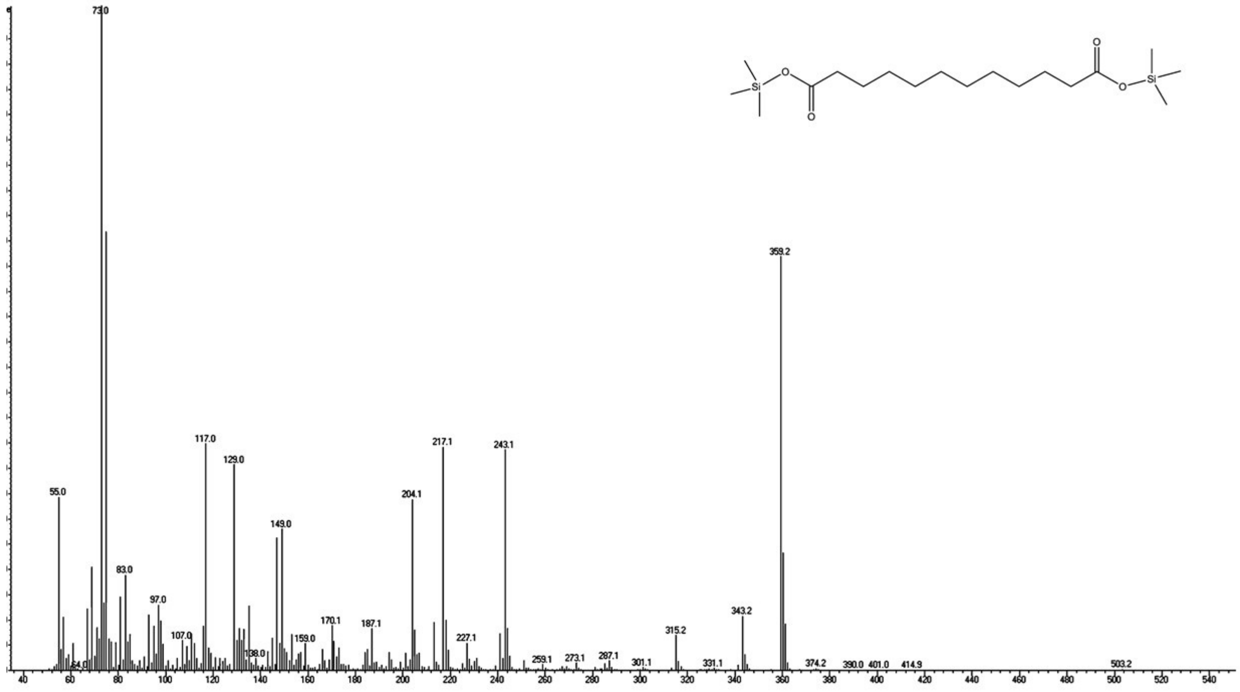

Figure 2S: DODA EI-GCMS spectrum as trimethylsilyl derivative.

Glucose uptake procedure:

Growing DMEM cells media were aspirated and cells were washed quickly with Dulbecco's phosphate-buffered saline (DPBS). New media with 5mM $^{13}C_6$-glucose was added. 150 µl aliquots were collected at time=0 and time=6 hours. To the 25µl of the cell media aliquot, 10 µl of 1mM galactose (Millipore Sigma) was added. $^{13}C_6$-glucose was extracted from media aliquots using 400

µl methanol/chloroform (1:1), followed by 400 µl of water. Samples were rocked for 10 minutes, then centrifuged at 5000 rpm for 5 minutes. The resulting polar upper phase was separated from the bottom nonpolar phase and dried down under nitrogen. Dried media extracts were derivatized using 0.2M hydroxylamine hydrochloride (Millipore Sigma) at 90°C for 40 minutes, followed by derivatization with acetic anhydride at 90°C for 60 min. The derivatized samples were dried down under nitrogen and re-suspended in ethyl acetate, transferred to a glass vial, and injected to GCMS. GCMS analysis was carried out with a 5977 GC-MS (Agilent) operated in EI mode and equipped with an HP-5ms column (Agilent). Retention times and fragmentation mass spectra of were confirmed with commercially available standards and the NIST library.

Oven program as following: initial oven temperature 80°C, hold for 3 minutes, then ramp 15°C/minute up to 190°C , ramp 5°C/minute up to 205°C, ramp 1°C/minute up to 212°C and finally ramp 15°C/minute up to 310°C hold for 2 minutes. The complex oven program is required to achieve maximum separation between structural isomers $^{13}C_6$-glucose and galactose (Figure S3). The following ions were monitored under single ion monitoring mode (SIM): m/z 319, for $^{13}C_6$-glucose, and m/z 314 for galactose.

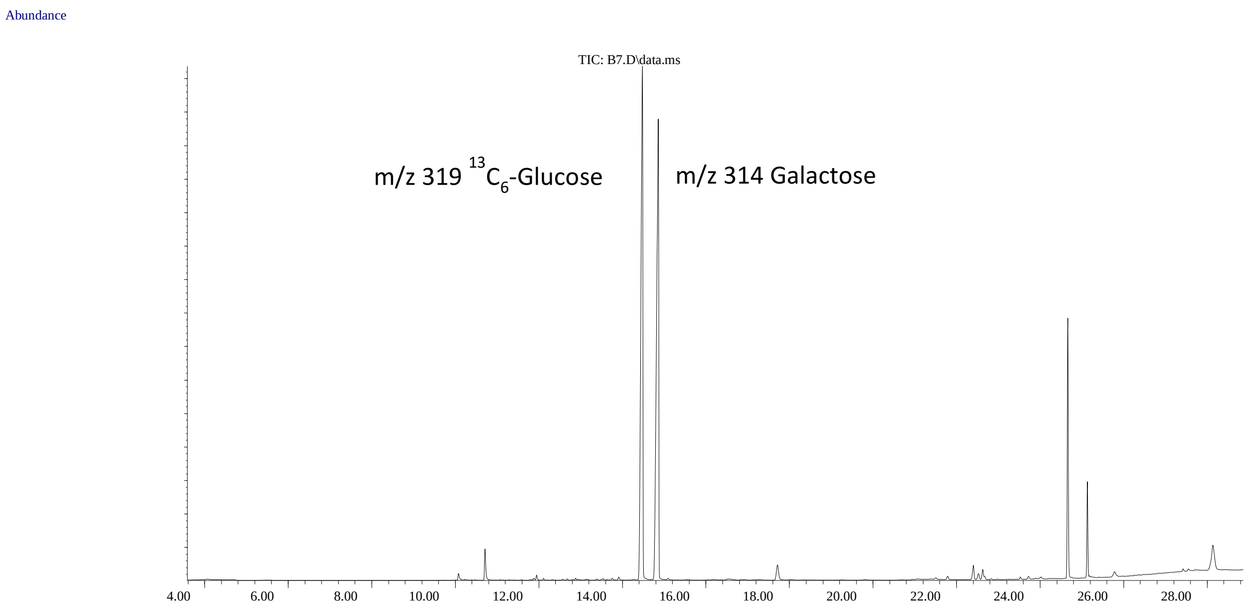

Figure 3S: Total ion chromatogram. EI-GCMS
